# Effect of formaldehyde and urea contaminated feed exposure into the liver of young and adult pigeons (*Columba livia*)

**DOI:** 10.14202/vetworld.2021.769-776

**Published:** 2021-03-26

**Authors:** Imam Hasan, Munmun Pervin, Md. Alamgir Kobir, Sakib Hossain Sagor, Mohammad Rabiul Karim

**Affiliations:** 1 Department of Anatomy and Histology, Faculty of Veterinary Science, Bangladesh Agricultural University, Mymensingh-2202, Bangladesh; 2 Department of Pathology, Faculty of Veterinary Science, Bangladesh Agricultural University, Mymensingh-2202, Bangladesh; 3 Department of Medicine, Faculty of Veterinary Science, Bangladesh Agricultural University, Mymensingh-2202, Bangladesh

**Keywords:** birds, formaldehyde, histopathology, liver, toxic food contaminants, urea

## Abstract

**Background and Aim:**

Nowadays, toxic chemical contaminants in food are a major food safety problem in Bangladesh. Among toxic food contaminants, formalin is used to preserve fruit, vegetables, and fish, where urea is used for the whitening of rice and puffed rice. The purpose of this study was to determine the biochemical and histopathological effects on the liver of young and adult pigeons after exposure to formalin and urea contaminated feed.

**Materials and Methods:**

A total of 15 young and 15 adult pigeons were divided into control group, formaldehyde exposed group (2.5 mL formalin/kg feed), and urea exposed (1 g/kg feed) group. Each group consisted of five pigeons. After the experimentation procedures, the blood samples were collected for biochemical study, and the liver tissue was collected for histomorphological study. The statistical analysis was performed using the Student’s t-test, and p<0.05 was considered as statistically significant.

**Results:**

The aspartate transaminase serum hepatic enzyme was significantly increased in both formalin and urea exposed young and adult pigeons than the control pigeons. In control pigeons, parenchymal hepatocytes and non-parenchymal cells are regularly arranged. However, histological observation of the liver of formalin and urea exposed young, and adult pigeons showed coagulation necrosis with infiltration of many inflammatory cells around the central and portal veins. The necrotic areas are more extensive with massive infiltration of inflammatory cells in the liver of formalin-treated pigeons than the urea treated pigeons.

**Conclusion:**

The present study results show that low concentrations of formalin and urea in feed induced liver lesions in pigeons in different extents and indicate that exposure to toxic chemicals may affect homeostasis of the liver and cause liver injury or act as a co-factor for liver disease.

## Introduction

Food safety issues have been one of the most commonly discussed issues in Bangladesh. The unhygienic practice of food handling is a key food safety issue. Unhygienic food is one of the main reasons for diarrheal diseases and malnutrition [1,2]. The use of harmful and poisonous food adulterators is another major concern for food safety in Bangladesh. Adulteration of food with toxic chemicals has reached an alarming level [3,4]. The variety of chemicals and coloring agents used in food is beyond imagination. For example, calcium carbide is applied on fruits to ripen, formalin on fish, fruits, milk, and vegetables as preservatives, urea to whiten rice and puffed rice, soap in ghee. Various coloring agents are used in sauces, juices, lentils, and oils. Calcium carbides are used for artificial fruit ripening, leading to cancer of various organs such as the liver, kidney, skin, prostate, and lungs. Food adulteration with formalin is a cold-blooded offence. Formalin is an aqueous solution of 37-40% of formaldehyde (FA). The extensive use of formalin in fruit, vegetables, fish, meat, and milk for long-term preservation creates a serious hazard to public health and nutrition at an alarming rate [5]. In the manufacturing, clothing, furniture, plastics, medicinal, cosmetic, and pharmaceutical industry, formalin is frequently used and has a significant impact on regular users [6]. Formalin is a toxic substance that is not safe for handlers. Exposure to formalin as a form of gas or vapor can irritate the eyes and nose, resulting in watery eyes, sneezing, headache, burning sensations in the throat, cough, and difficulty breathing [7]. However, FA is produced extracellularly and exists in small amounts in all body tissue [8,9]. It is produced through enzymatic oxidative demethylation of DNA, RNA, and proteins in the myeloperoxidase reaction of activated neutrophils or monocytes [10]. Due to its water solubility and reactivity, FA can be easily dispersed in many cells and tissues of the human body [11,12]. FA induces inflammation of the lining of the mouth, throat, gastrointestinal tract, subsequent ulceration, and necrosis of the mucous lining of the gastrointestinal tract, causing parenchymatic organ lesions [13]. However, FA has a detrimental effect on biological systems. FA-exposition can cause irritation, nasopharyngeal cancer, and leukemia in the eye and upper respiratory tract [14]. It causes various histopathological changes that indicate the damage of the liver tissue, focal hepatic necrosis, hepatic enlargement, decreased weight, and degeneration of hepatocellular fatty that is related to the length of the period of exposure [15,16]. Formalin intake can lead to kidney disease, liver cirrhosis, cancer, and respiratory disease. It causes harm to the reproductive system by inducing oxidative stress [16,17]. Once formalin is added, it is said that it cannot be removed entirely. Hence, washing of fruit and vegetable is not sufficient to make it safe. Intake of formalin over a long period may cause respiratory, digestive, cardiac, nephrological, and neurological problems alongside cancer [2,14,18]. FA toxicity has been reported in multiple tissues or organs (the liver, heart, brain, lung, and gonads) of the exposed rats, mice, and pigeons [17,19-21]. Karim *et al*. [21] have shown that FA-contaminated feed exposure causes degeneration of spermatogenic cells in the testis of adult pigeons. However, FA has a harmful effect on the biological system of mammals [17]. Higher exposures to formalin can cause significant inflammation of the lower respiratory tract, leading to hemorrhagic to fibrotic changes in humans [22]. Rats inhaled 20.3 ppm FA gas for 13 weeks, 8 h/day, 5 days a week, induced various toxic effects in the liver tissue such as hepatic enlargement, hepato-cellular fatty degeneration, and hepatic necrosis [16]. An experimental study on rat kidney showed that rat exposure to FA vapor in the concentration of 1.5 ppm 4 h/day, for 4 days/week for 18 weeks led to mild congestion in the glomeruli, focal congestion, and vacuolar (hydropic) degeneration of tubular cells, and necrosis [23]. Broiler chicks administered 5 mL or more formalin/kg feed for 8 weeks had decreased feed intake and body mass, focal necrosis, and petechial hemorrhages in crops and intestines [24]. Urea is highly toxic to the human body that can cause cancer and multiple ulcers [25]. Rice is adulterated with urea for whitening. Puffed rice (muri) is adulterated with hydrose (sodium hydrosulfite) and urea to make it whiter and bigger. Urea is also harmful to the liver and kidney [26-28].

The use of toxic contaminants or adulterants in foods and food products may cause a potential threat to the health of livestock and human beings. The consumption of toxic contaminants or food adulterants must be involved as necessary co-factors in the pathogenesis of vital organ injury. FA is rapidly absorbed by the nasal passages, digestive tract after ingestion, and severe adverse effects. The digestive system is the most important target of formalin. However, no comprehensive study on the histomorphological alteration of the liver in formalin and urea exposed birds has not yet been undertaken. Therefore, it is essential to know the toxic effects of formalin and urea contaminated feed on the liver tissues of pigeons.

Therefore, the present study was carried out to evaluate the biochemical and histomorphological changes in the architecture of the liver of young and adult pigeons after FA and urea exposure.

## Materials and Methods

### Ethical approval

The present study was approved by the Animal Welfare and Experimentation Ethics Committee, Bangladesh Agricultural University, Mymensingh, Bangladesh (Protocol Number: AWEEC/BAU/2019-40).

### Study location and period

The research work was conducted in the Department of Anatomy and Histology, Faculty of Veterinary Science, Bangladesh Agricultural University, Mymensingh-2202 and Village-Roholy, Saturia, Manikganj-1800, Bangladesh from July 2018 to June 2019.

### Animals and experimental procedures

The experimental animals used in this study were 15 young (18-days-old age; 136-166 g) and 15 adults (>12-month-old age; 203-215 g) healthy pigeons (*Columba livia*). The pigeons were housed in cages and fed a standard diet (mastered, rice, sesame, and wheat) twice daily and filtered tube-well water *ad libitum*. After 1-week acclimatization, the pigeons were categorized as the control group, FA exposed, and urea exposed group (each group consisted of five pigeons). Five young and five adult pigeons were fed an FA-contaminated feed daily in the morning and fed a standard diet daily evening for 7 days. The previous studies have shown that the 2.5 mL formalin/kg feed in broiler chicken, Japanese quails, and pigeons has no adverse effect on body weight and feed intake that suggestive of a beneficial dose [21,24,29]. In the experiment, this dose was used. Formalin (40% aqueous solution of stock FA powder) was appropriately mixed with pigeon feeds (2.5 mL formalin or 1000 mg/kg feed). Five young and five adult pigeons were fed urea contaminated feed at a rate of 1 g urea/kg feed daily in the morning and fed a standard daily diet for 7 days in the evening. The other five young and five adult pigeons were used as the control and fed a standard diet twice daily (morning and evening). After 7 days of FA and urea exposure (in the morning of the 8^th^ day), all pigeons were weighed and euthanized under deep anesthesia using 5 mL chloroform-soaked cotton in the vacuum glass chamber for 2-3 min. After proper anesthesia, a post-mortem examination was carried out.

### Serum enzyme analysis

The blood was collected from the heart. The blood containing the test tube was placed in a slanting position at room temperature (32°C) for 30 min. The serum was separated from the clotted blood by centrifugation at 3000 rpm for 20 min and again for 10 min. The supernatant was then collected by micro-pipette in the Eppendorf tube and stored in the refrigerator at -20°C until analysis of aspartate transaminase (AST). The serum test was evaluated by spectrophotometry using commercially available kits (Randox Laboratories, Crumlin, UK) following the manufacturer’s instructions.

### Collection of the liver for histopathology

Following deep chloroform anesthesia, the internal visceral organs, including the liver, was visualized through the ventral mid-line thoracoabdominal opening, and the gross anatomy was observed very carefully. Then, the liver was removed, and its color and gross texture were observed. The weight of the liver was measured and recorded. Subsequently, the liver tissues from the different lobes were collected and immediately fixed to 10% neutral buffered formalin (NBF) by standard method. Then, NBF-fixed tissues were dehydrated with ascending graded alcohols and embedded in paraffin and lastly sectioned at 6 μm in thickness using a sliding microtome (Euromax^R^, Japan). The deparaffinized sections were stained with hematoxylin and eosin (H and E) for histopathological examination. The formalin and urea induced histopathological changes were studied using a light microscope (LABOMED, Labo America Inc., CA 94538).

### Statistical analysis

All the obtained data were analyzed and statistically evaluated by Student’s t-test using SPSS, version 18.0 software (IBM Corp., NY, USA). p≤0.05 was considered statistically significant. All the results were expressed as mean ±SE.

## Results

### Effects on body weight, relative organ weight, and gross morphology of liver of pigeons

**Figure-1:**
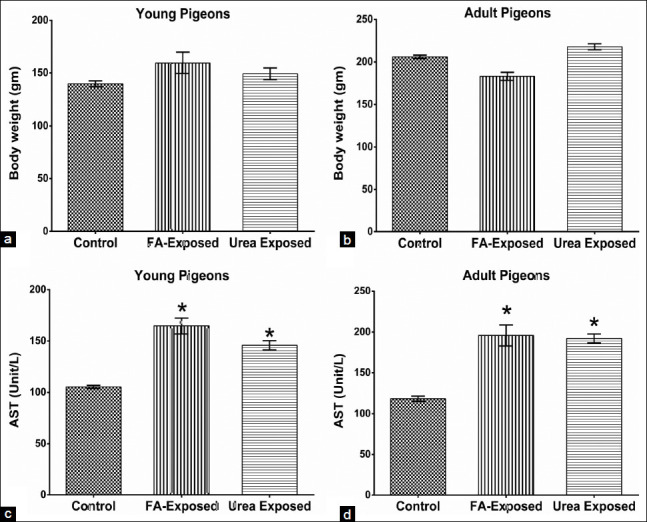
Effect of administration of formaldehyde (FA) and urea contaminant food on the body weight and level of aspartate aminotransferase (AST) serum enzyme in young and adult pigeons. (a) Effect on the bodyweight of young pigeons. (b) Effect on the bodyweight of adult pigeons. No significant changes in body weight were seen in FA and urea exposed young and adult pigeons as compared to the control. (c) Effect on the serum AST level of young pigeons. (d) Effect on the serum AST level of adult pigeons. AST level was significantly increased in FA and urea exposed pigeons in comparison with control. Student’s t-test between each group at each time-point. ∗p<0.05 control versus FA and urea exposed pigeons. FA=Formaldehyde.

**Figure-2:**
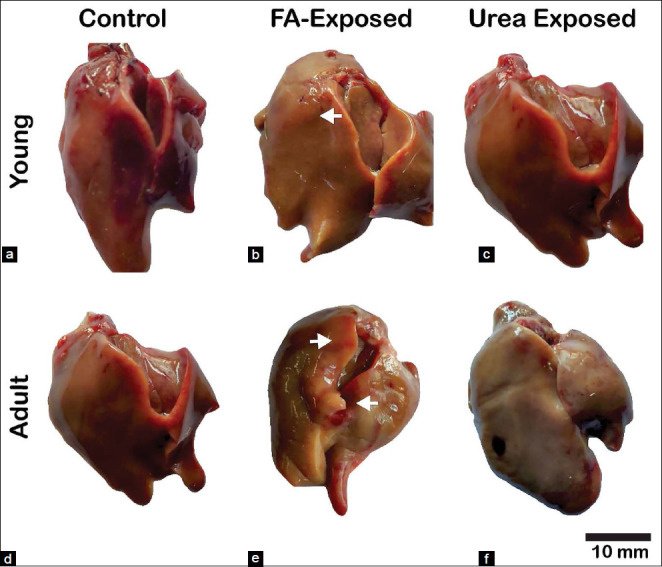
Effect of administration of formaldehyde (FA) and urea contaminant feed on liver morphologies in the young and adult pigeons. (a and d) Control young and adult pigeons’ liver. No congestion and hemorrhage are found in those groups. (b and e) young and adult pigeon’s liver intoxicated with FA: Gross image shows pale color with petechial hemorrhage (white arrow) on the parietal and visceral surfaces with a large discoloration area. (c) Young pigeon liver treated with urea: appeared brownish. (f) Adult pigeon liver treated with urea: Was pale and slightly yellowish. FA=Formaldehyde. Scale Bar=10 mm.

This study found that there is no significant (p>0.05) difference in body weight gain among FA and urea treated young and adult pigeons (Figures-1A and 1B). The relative organ weight indicated that the weight of the liver of FA and urea exposed young and adult pigeons were not significantly different from control pigeons. The color, size, and shape of the liver of both young and adult control pigeons were normal (Figures-2A and 2D). There were no changes in the size and shape of the liver of FA and urea treated pigeons. However, the liver revealed pale color with petechial hemorrhage on the parietal and visceral surfaces in FA-exposed young and adult pigeons (Figures-2B and 2E). The liver of urea exposed adult pigeon showed pale and slightly yellowish color, whereas, in young pigeons, the liver was normal (Figures-2C and 2F).

### Effects on serum hepatic marker enzyme

The high levels of aspartate aminotransferase (AST) hepatic serum marker reflected hepatotoxicity in both formalin and urea treated exposed pigeons compared to the control. In the present study, there was a significant increase (p<0.05) in the AST levels after the administration of FA and urea in young and adult pigeons as compared to the control group (Figures-1C and 1D). The serum levels of AST indirectly reflect liver injury.

### Histopathological changes in the liver tissue

**Figure-3:**
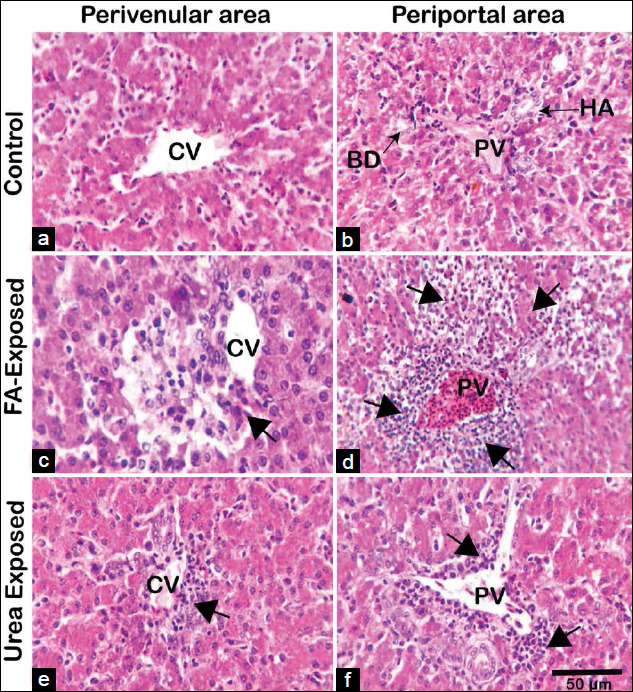
Histopathology of the liver of control, formaldehyde (FA)- and urea-exposed adult pigeons (hematoxylin and eosin). (A and B) Photomicrograph from the control group’s liver sections shows healthy hepatic cells with normal cellular architecture, central-portal veins, and apparent hepatic cell cord in young pigeons. The HA and the BD are also seen. (C and D) Photomicrograph from FA treated adult pigeon showing severe necrosis with mononuclear cellular infiltration surrounding the central vein (arrowhead), massive hepatocytic necrosis comprising coagulation necrosis, and apoptotic hepatocytes around the congested portal vein (arrowhead). (E and F) Urea treated young pigeon, mild coagulation necrosis with infiltration of a small number of inflammatory cells around the central and portal veins of adult liver were seen. The necrotic areas are more extensive with massive infiltration of inflammatory cells in the liver of FA-treated pigeons than the urea treated pigeons. FA=Formaldehyde, CV=Central vein, PV=Portal vein, BD=Bile duct, HA=Hepatic artery. Scale Bar=50 mm.

**Figure-4:**
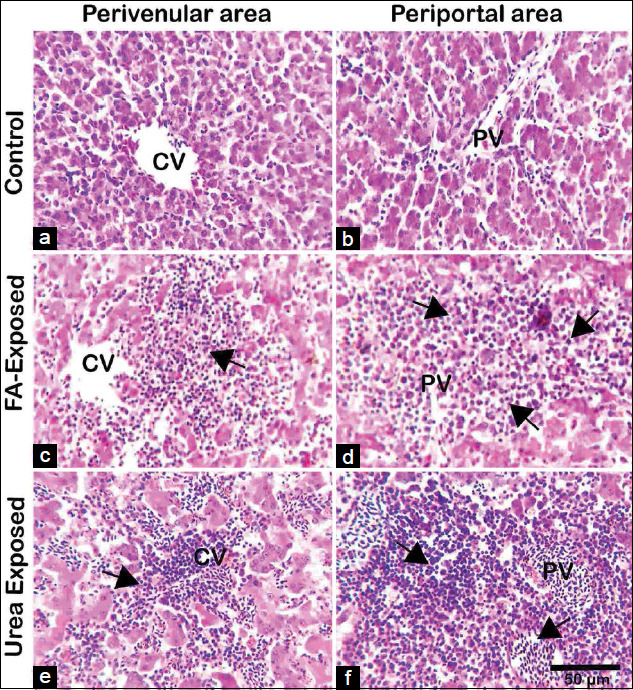
Histopathology of the liver of control, formaldehyde (FA) - and urea-exposed young pigeons (hematoxylin and eosin). (A and B) Photomicrograph from the control group’s liver sections shows healthy hepatic cells with normal cellular architecture, central-portal veins, and apparent hepatic cell cord in young pigeons. (C and D) Photomicrograph from FA treated young pigeon showing severe necrosis with mononuclear cellular infiltration surrounding the central vein (arrowhead), massive hepatocytic necrosis comprising coagulation necrosis, and apoptotic hepatocytes around the congested portal vein (arrowhead). (E and F) Urea treated young pigeon; sinusoids were congested and dilated with severe coagulation necrosis and infiltration of many inflammatory cells around the young liver’s central and portal veins. FA=Formaldehyde, CV=Central vein, PV=Portal vein, Scale Bar=50 mm.

H and E stained sections of the liver of control pigeons showed normal hepatic cord, hepatocytes with acidophilic granular cytoplasm, and central vesicular nuclei as well as well-preserved cytoplasm and well-defined nucleus. The portal vein and the bile ducts were not dilated or enlarged. The arrangements of parenchymal hepatocytes as hepatic cords and non-parenchymal cells are regular in form. There were no signs of necrosis or cellular damage in control pigeons (Figures-3A and B and 4A and B). However, in FA treated young and adult pigeons liver showed, severe coagulation necrosis with infiltration of inflammatory cells surrounding the central and portal veins (Figures-3C and D and 4C and D). The sinusoids were congested and dilated with mononuclear cellular infiltration due to coagulative necrosis, hemorrhage, and apoptotic hepatocytes around the congested portal vein in FA exposed adult pigeon (Figures-3C and D and 4C and D). The liver lesions were more predominant in FA exposed adult pigeons compared to the liver of young pigeons.

The liver of urea-exposed young pigeons showed that sinusoids were congested and dilated with severe coagulation necrosis and infiltration of inflammatory cells around the central and portal veins (Figures-3E and F). Mild coagulation necrosis with infiltration of inflammatory cells around the central and portal veins were seen in the liver of urea-exposed adult pigeon. (Figures-4E and 4F). The lesions of the urea exposed young pigeons were severe compared with the FA treated adult liver. The liver of young birds was susceptible to FA and urea exposure. The necrotic areas were extensive with massive infiltration of inflammatory cells in FA-treated adult pigeons’ liver than the urea treated adult pigeons.

## Discussion

The liver plays a crucial function in carbohydrate, protein, lipid, and amino acid metabolism in the body. The liver also makes various processed metabolites available to other tissues for bioavailability [9]. FA is known as poisonous at a particular concentration. The risk of adverse effects is increased at room temperature due to its volatility. All those working closely with it are concerned about the toxicity of FA. Embalmers, anatomists, technicians, and medical or veterinary students are among the people who have high exposure to FA [11]. Therefore, FA intoxication at different doses and routes affect the metabolic pathway of the liver. In the present study, gross, biochemical, and histomorphological studies have been carried out to investigate the changes in the liver of young and adult pigeons after FA and urea exposure.

In the present study, no significant difference was found in the bodyweight of both FA- and urea exposed young and adult pigeons indicating that no harmful impact on weight gain in short time exposure. This result was similar to our previous report on adult pigeons [21]. However, when male Japanese quails fed 10 mL formalin/kg feed for 8 weeks, there was a decrease in feed intake and body weight gain [29]. Babar *et al*. [24] reported a decreased body mass when broilers fed 5 mL formalin/kg feed for 8 weeks. No difference in growth and feed efficiency in chickens fed 0.3% FA in the feed reported by Spears *et al*. [30]. The serum enzyme AST threshold in FA and urea exposed pigeons was significantly increased in both young and adult pigeons compared to control pigeons. These results showed hepatic injury and disturbance of body homeostasis induced by exposure to FA and urea. Similarly, Karim *et al*. [21] reported that FA-contaminated feed exposure significantly elevated serum enzyme AST in adult pigeons. However, there have been no available reports on young birds. Serum enzymes AST and ALT were significantly increased when adult mice exposed to FA [31]. It was reported that damage to hepatocytes, intrahepatic, and extrahepatic bile ducts were caused by FA [29]. This is due to the metabolic reaction. Because FA is an essential metabolic intermediate in all cells and in the biosynthesis of purine, thymidine, and certain amino acids in all animal species [30]. The level of endogenous FA is maintained at a low concentration under physiological conditions, regulated by the expression and activity of both FA-generating and FA-degrading enzymes [30]. When ingested, free and reversibly bound FA is readily absorbed into the gastrointestinal tract and joins the endogenous FA pool [9]. FA is rapidly oxidized by NAD-dependent FA dehydrogenase through a glutathione (GSH)-dependent mechanism into formic acid in the blood and liver. Formic acid, in turn, enters the body’s carbon pool or is further oxidized to carbon dioxide and water in the liver and erythrocytes. This reaction occurs more slowly in humans than in dogs or rodents. Residual formic acid and other minor metabolites that are not metabolized are excreted through urine, feces, or expired air [11] and the relative amounts depending on the route of administration [5,8,10]. FA is generally present in reversibly and irreversibly bound forms due to its chemical reactivity, as free FA, comprising 1-2% of the overall measurable concentrations in tissues, and as irreversibly bound FA to proteins and nucleic acids, representing between 50% and 80% of endogenous FA [10]. General symptoms of toxicity arise as exposure conditions (e.g., food and drinking water concentrations) lead to local lesions, which ultimately affect the overall health of the animals exposed. This applies to the hepatotoxic effects following *in vivo* exposure [20].

Grossly the liver of FA-exposed young and adult pigeons revealed pale color with petechial hemorrhage on the parietal and visceral surfaces, wherein urea-exposed adult pigeons showed pale and slightly yellowish color. Similar liver lesions have also been reported in rats following oral administration of FA [31]. Short-term exposure to FA encourages oxidative damage and inflammation of adult rats’ trachea and diaphragm muscles, causing metaplasia, ulceration, and increased mucus [32]. In histopathology of the liver, the present study revealed that FA exposure induced severe histopathological changes in pigeons’ livers. There are no reports about formalin and urea induced pathological changes in the liver of pigeons or any other birds. Bakar *et al*. [33] reported that the loss of cytoplasm, vacuolization, pyknotic nuclei, and mononuclear cells’ infiltration was detected in rat liver cells exposed to FA. In addition, FA had various impacts on the respiratory tract, lung, eye, and gonad [21]. In the present study, FA exposed to both young and adult pigeons revealed massive coagulation necrosis around the congested portal vein, including inflammatory cell influx, hemorrhage, and apoptotic hepatocytes. However, the present results were consistent with that of other FA exposure research on rats, although the dosage, duration, and route of administrations of FA were different [16,34]. Theprevious studies demonstrated that exposure to FA could cause hepatic necrosis, and hepatocellular fatty degeneration was observed in the livers of the mice exposed to 0.08 mg/m^3^ FA for 3 days [16,34]. However, when the exposure time was extended to 7 days presenting loss of the cytoplasm in some hepatocytes, suggesting FA inhalation could induce subtle injury to liver cells [35]. Severe liver alterations, including hepatocyte degeneration and disorganization, partial loss of hepatocyte cytoplasm, and cytoplasmic vacuoles were identified in animals exposed to FA. Besides, vena centralis and sinusoids have been found to enlarge [36] substantially. In 1981, at the Battelle Norwest Laboratory mice exposed 40 ppm of FA gas for 6 h a day, 5 days a week, and 13 weeks declared focal necrosis in liver cells [10]. The animals were introduced to FA (200 mg/m^3^ over 122 days). They found mononuclear cell infiltration and inflammatory effects in the liver specimens by Wooster *et al*. [37], which were reproduced in the present study following FA contaminated feed exposure in the liver of young and adult pigeons. No data were found in the literature about the effects of FA-induced hepatotoxicity in the pigeon.

Chemicals used in foodstuffs are potent carcinogens that cause cancer in many tissues of the human body [37,38]. Urea adulteration in puffed rice processing has become a concerning issue in the general people of Bangladesh. Urea generally has low toxicity in humans as it is an excreted metabolite through prolonged ingestion of high levels of urea that may develop and worsen pathological conditions [25,28]. Urea added rice to make it whiter and added to Muri (puffed rice) to make it whiter and puffier. The liver of young pigeons treated with urea showing the inflammatory cells infiltration surrounding the central and portal vein with dilation of the portal vein wherein adult pigeons treated with urea produced severe hepatic damage, including the extensive degeneration of hepatocytes with necrosis, inflammation, and inflammatory cell infiltration around the perivenular and periportal area. Few researches have already been conducted on various animals (Cynomolgus monkeys, rats, and hamsters, etc.) about the hazardous effects of FA and urea through various exposure routes [39-41]. However, no research on the impact of FA and urea on the liver of avian species have been available.

## Conclusion

The present results have suggested that the toxic feed contaminant use of FA and urea is detrimental to health and induces severe effects on the liver histology of young and adult pigeons. Particularly, exposure of FA (2.5 mL formalin/kg feed) and urea (1 g urea/kg feed) through feedstuff causes the extensive degeneration of hepatocytes, coagulation necrosis with infiltration of a large number of inflammatory cells around the perivenular and periportal area with dilation of the portal vein of the liver. The present findings will help anatomists, pathologists, medical and veterinary students are careful about the detrimental consequences of FA and urea exposure as well as to help development of antidote against FA- and urea-base hepatotoxicity. Therefore, it is very alarming if the human being exposed to this toxic food contaminant may cause liver injury. To prevent these indictable offenses of adding formalin and urea to foodstuffs, strict laws should be established.

## Authors’ Contributions

MRK and MP designed the experiment. IH, MAK, SHS, MP and MRK undertook the experiment. IH, MP, SHS, and MRK interpreted the results. MRK and IH wrote up the draft. MRK and MP checked the manuscript critically. All authors read and approved the final manuscript.
